# Laser-light cueing shoes with integrated foot pressure and inertial sensing for investigating the impact of visual cueing on gait characteristics in Parkinson’s disease individuals

**DOI:** 10.3389/fbioe.2024.1334403

**Published:** 2024-01-31

**Authors:** Hsiao-Lung Chan, Rou-Shayn Chen, Cheng-Chung Kuo, Yi-Tao Chen, Jiunn-Woei Liaw, Guo-Sheng Liao, Wan-Ting Lin, Shih-Hsun Chien, Ya-Ju Chang

**Affiliations:** ^1^ Department of Electrical Engineering, Chang Gung University, Taoyuan, Taiwan; ^2^ Department of Biomedical Engineering, Chang Gung University, Taoyuan, Taiwan; ^3^ Neuroscience Research Center, Chang Gung Memorial Hospital Linkou, Taoyuan, Taiwan; ^4^ Department of Neurology, Chang Gung Memorial Hospital Linkou, Taoyuan, Taiwan; ^5^ School of Medicine, College of Medicine, Chang Gung University, Taoyuan, Taiwan; ^6^ Department of Mechanical Engineering, Chang Gung University, Taoyuan, Taiwan; ^7^ Center for Advanced Molecular Imaging and Translation, Chang Gung Memorial Hospital Linkou, Taoyuan, Taiwan; ^8^ School of Physical Therapy and Graduate Institute of Rehabilitation Science, College of Medicine, and Health Aging Research Center, Chang Gung University, Taoyuan, Taiwan

**Keywords:** Parkinson’s disease, laser-light visual cueing, one-side cueing at a time, foot pressure, inertial sensing, gait characteristic

## Abstract

Gait disorders are a fundamental challenge in Parkinson’s disease (PD). The use of laser-light visual cues emitted from shoes has demonstrated effective in improving freezing of gait within less restrictive environments. However, the effectiveness of shoes-based laser-light cueing may vary among individuals with PD who have different types of impairments. We introduced an innovative laser-light visual shoes system capable of producing alternating visual cues for the left and right feet through one-side cueing at a time, while simultaneously recording foot inertial data and foot pressures. The effects of this visual cueing system on gait patterns were assessed in individuals with PD, both those with well-gait and those with worse-gait. Our device successfully quantified gait characteristics, including the asymmetry in the center of pressure trajectory, in individuals with PD. Furthermore, visual cueing prolonged stride times and increased the percentage of stance phase, while concurrently reducing stride length in PD individuals with well-gait. Conversely, in PD individuals with worse-gait, visual cueing resulted in a decreased freeze index and a reduction in the proportion of intervals prone to freezing episodes. The effects of visual cueing varied between PD individuals with well-gait and those with worse-gait. Visual cueing slowed down gait in the well-gait group while it appeared to mitigate freezing episodes in worse-gait group. Future researches, including enhancements to extend the projection distance of visual cues and clinical assessments conducted in real-world settings, will help establish the clinical utility of our proposed visual cueing system.

## 1 Introduction

Gait disturbances constitute a central challenge for individuals with Parkinson’s disease (PD). These challenges encompass issues like bradykinesia, shuffling steps, festinating gait, and freezing of gait (FOG), ultimately contributing to postural instability and a higher susceptibility to falls. A potential remedy involves incorporating cueing supports through auditory, somatosensory, and visual inputs. Auditory cueing is frequently employed through the delivery of rhythmic auditory stimulation, leading to a notable enhancement in both stride length (Suteerawattananon et al., 2004; Lee et al., 2012) and turn speed (Nieuwboer et al., 2009), along with a reduction in the number of freezing episodes (Lee et al., 2012) among individuals with PD. Additionally, the application of rhythmic auditory cueing resulted in fewer deviations in cadence during extended periods of walking (Ginis et al., 2017). Furthermore, closed-loop auditory feedback, achieved by translating steps into cueing clicks, demonstrated improvement in walking speed and stride length among individuals with PD (Baram et al., 2016).

On the other hand, somatosensory cueing has proven effective in enhancing gait performance among individuals with PD. One notable approach involves applying rhythmic sensory electrical stimulation to the waist, resulting in a significant reduction in the time required to complete a walking task (Rosenthal et al., 2018). While numerous approaches rely on vibration stimulation, this method stands out for its notable benefits. For example, the application of rhythmic vibration stimulation to the wrist resulted in a significant increase in both stride time and step length (van Wegen et al., 2006). Similarly, alternate rhythmic vibration to the lower legs on both sides led to a significant increase in stride length, cadence, and velocity (De Nunzio et al., 2010). Additionally, the implementation of either rhythmic vibration stimulation or FOG-initiated vibration cueing on the lower leg significantly improved gait task completion time (Li et al., 2023). Furthermore, delivering vibration stimulation to the wrist during the stance phase of gait significantly reduced the percentage of freezing time and the FOG index during turning (Mancini et al., 2018). Moreover, step-synchronized vibration stimulation of the soles significantly increased walking speed, stride time, stride duration, and cadence (Novak and Novak, 2006).

In contrast to auditory and somatosensory cueing, visual cueing involves providing a visible type of stimulation in front of individuals. Conventional visual cues typically involve placing transverse lines or inverted sticks along the walking pathway. Notably, this approach has been demonstrated to increase stride length and velocity (Azulay et al., 1999; Suteerawattananon et al., 2004; Lee et al., 2012), ameliorate gait abnormalities and mitigate the frequency and duration of FOG episodes among individuals with PD (Nonnekes et al., 2015).

Nonetheless, the real-world implementation of visual markers along walkways is constrained by its effectiveness within specific environments. In response to this challenge, an innovative solution that involves utilizing specialized equipment, such as a cane (Bryant et al., 2010) or an assisted walker equipped with laser light devices (Cubo et al., 2003; Wu et al., 2020) is used to project guiding lines onto the ground, offering an immediate direction for individuals afflicted by PD to step over, irrespective of the available space. Moreover, these methodologies have demonstrated a short-term effect, including an increase in stride length and a decrease in freezing episodes (Bryant et al., 2010), as well as long-term efficacy in reducing FOG questionnaire scores and the occurrence of falls when compared to a baseline period of using canes or walkers without visual cues over a 1–2 month period (Donovan et al., 2011).

Additionally, innovative wearable laser cueing devices were strategically placed at the chest level (Lewis et al., 2000; Velik et al., 2012) or waist level (Cao et al., 2020; Suputtitada et al., 2022; Zhang et al., 2023), projecting a laser beam onto the ground, creating an extended visual cue in front of individuals. Laser-light cueing at chest level has shown significant benefits in individuals with PD, including increased stride length and gait velocity (Lewis et al., 2000), reduced duration and number of freezing episodes (Velik et al., 2012), and improved speed of turning (Nieuwboer et al., 2009) when compared to a baseline condition without visual cues. Moreover, laser-light cueing at waist level has also demonstrated notable advantages, including increased stride length and gait velocity (Suputtitada et al., 2022; Zhang et al., 2023), longer step length (Cao et al., 2020), an enhanced proportion of the double stance phase (Zhang et al., 2023), compared to periods without visual cues. Additionally, when combined with a plantar pressure plate positioned at the midpoint of the walkway, a significant reduction in the anteroposterior distance from the center of both feet during the double stance phase was observed, indicating improved gait stability through the use of the visual cue (Zhang et al., 2023).

Considering that individuals commonly wear shoes during their daily activities, especially outdoors, integrating a laser-light visual cueing device into footwear emerges as a practical and convenient solution for real-life scenarios. By incorporating a laser light device on each foot, projecting two distinct laser beams that interlace, individuals can be prompted to step over contralaterally (Zhao et al., 2013; Ferraye et al., 2016). Barthel *et al.* provided evidence that the application of visual cueing through laser-light shoes notably curtailed the occurrence of FOG episodes and the proportion of frozen time in individuals with PD. However, this application did not yield significant alterations in gait measures. (Barthel et al., 2018).

The footwear approach provides the added advantage of creating distinct cueing lines for both left and right side stepping, enhancing its versatility and adaptability. In the earlier stages of development, the laser was triggered during the stance phase using either a switch (Ferraye et al., 2016) or a pressure sensor membrane (Zhao et al., 2013) positioned beneath the sole of the opposite foot. This design allowed for autonomous activation of the laser light on either side. However, this setup carried the possibility of concurrent activation of visual signals from both the left and right sides when both feet were in contact with the ground. This scenario often arose during activities such as gait initiation, shuffling steps, and instances of FOG.

In this research endeavor, we conceptualized footwear integrated with laser-light visual cues, featuring an incorporation of 11 force-sensitive resistors (FSRs) within the insole for precise foot pressure assessments. Leveraging the captured foot pressure data, a real-time algorithm based on the center of pressures (COPs) from the left and right feet was developed. This algorithm facilitated the sequential activation of laser-light cues on a single side, thus preventing simultaneous cues.

We further enhanced our design by incorporating an inertial measurement unit (IMU) comprising an accelerometer, gyroscope, and magnetometer. This IMU, in conjunction with foot pressure data, enabled the computation of essential gait parameters such as stride time, stride length, and the percentages corresponding to stance, swing, single support, and double support phases. These parameters proved instrumental in the characterization of distinct gait patterns. Furthermore, the dataset encompassed metrics related to the COP, such as gait line length, single support length, anteroposterior position, and lateral symmetry, all of which could be assessed during ambulation. Additionally, the accelerometric data proved invaluable for quantifying the FOG index, a crucial parameter frequently used in the assessment of PD (Moore et al., 2008; Moore et al., 2013).

With these comprehensive measures in place, our investigation delved into the effects of laser-light cueing on gait adaptations and stability within two distinct PD groups, each exhibiting varying degrees of gait impairment–one group demonstrating well-preserved gait, while the other exhibiting more pronounced gait deficits.

## 2 Materials and methods

### 2.1 Laser-light visual-cueing shoes

**FIGURE 1:**
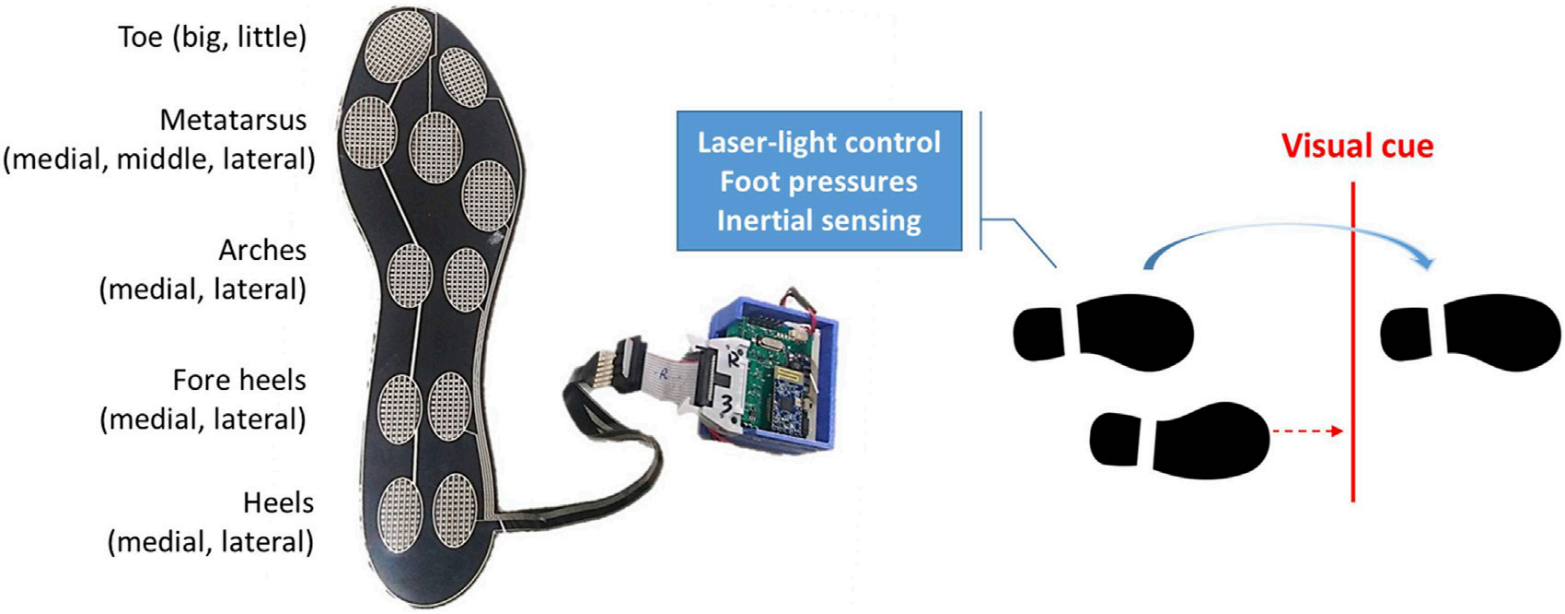
A laser-light control device with parallel foot pressures and inertial sensing.

We have created shoes that use laser-light technology to generate lines on the floor as visual cues, while simultaneously collecting foot inertial data and foot pressure information. As depicted in Figure 1, each shoe has a laser-light emitter on the top cap and a laser-light control device with parallel foot pressure and inertial sensors on the lateral side. The left shoe detects foot pressure to trigger visual cues for the right foot, while the right shoe detects foot pressure to trigger visual cues for the left foot. The forward distance of the laser line is configured to be 30–35 cm in front of the standing foot.

We utilized an IMU from STMicroelectronics (LSM9DS1, Geneva, Switzerland) to collect inertial data which consisted of a tri-axial accelerometer with a full-scale range of ±8 g, a tri-axial gyroscope with a full-scale range of ±500°/s, and a tri-axial magnetometer with a full-scale range of ±12 G. To capture foot pressures, we employed a custom-designed insole fitted with eleven FSRs from UNEO Incorporated (New Taipei City, Taiwan), strategically positioned at various locations on the foot including the big toe, little toe, medial, middle, and lateral metatarsus, medial and lateral arches, medial and lateral fore heels, and medial and lateral heels. These FSRs were manufactured using processing and printing-based micromachining technology, employing a resistance-type piezo-resistive polymer composite. Each FSR had a sensing range of 1–5 kg/cm^2^ and was individually calibrated using elastic-film pressurization to minimize resistance variance among the sensors (Chan et al., 2023). The custom insole had dimensions of 260 mm in height, 85 mm in metatarsus width, 55 mm in heel width, and a thickness of 0.63 mm. Therefore, we only included participants whose foot size matched the size of the customized insole as closely as possible.

A microcontroller (M451RG6AE, Nuvoton Tech. Corp., Hsinchu, Taiwan) utilizing the ARM Cortex-M4 architecture received digital data from the IMU at a sampling rate of 100 Hz through a serial peripheral interface bus. The microcontroller also converted the transformed voltages from eleven FSRs into digital data using an internal 12-bit analog-to-digital converter, operating at the same sampling rate. All the acquired data samples were wirelessly transmitted to a smartphone using a BLE 4.2 Bluetooth module (JDY-18, Shenzhen Innovation Technology, Shenzhen, China), while the smartphone simultaneously recorded video images.

The calculation of the COP involved evaluating eleven individual foot pressures, denoted *fp*_1_(*i*), *fp*_2_(*i*), …, *fp*_11_(*i*). COPx and COPy were determined by summing the products of the individual foot pressures and their corresponding coordinates, and then dividing by the total sum of the eleven foot pressures:

$$\text{COPx}\left(i\right)=\frac{\left.\sum \left({fp}_{k}\left(i\right)\right.*x_{k}\right)}{\sum {fp}_{k}\left(i\right)}$$

$$\text{COPy}\left(i\right)=\frac{\left.\sum \left({fp}_{k}\left(i\right)\right.*y_{k}\right)}{\sum {fp}_{k}\left(i\right)}$$

where *x*_*k*_ and *y*_*k*_ represent the centroid position of the *k*th FSR with respect to the local reference frame.

**FIGURE 2:**
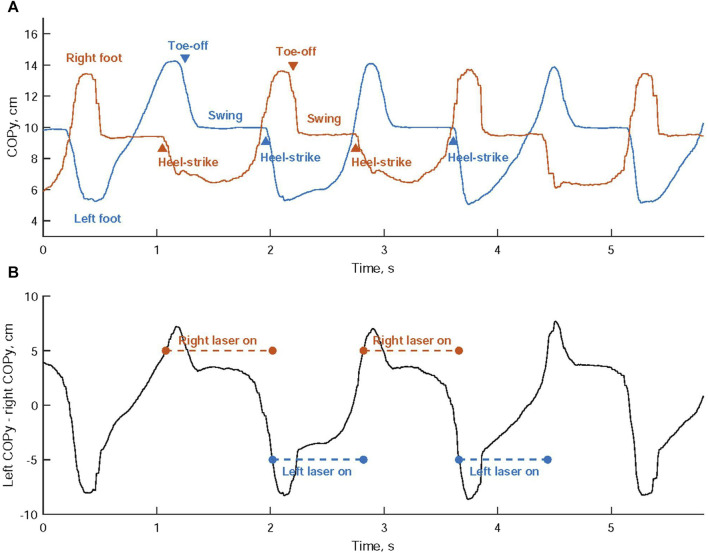
**(A)** The vertical trajectory analysis of the center of pressure (COP) is conducted for the movement of each foot. **(B)** Activation of the laser cues is determined by comparing the disparity in COP values between the left and right foot against predefined thresholds.

Laser light activation during walking was achieved through a one-sided cueing approach, wherein the laser light on either the left or right shoe was activated based on the COP coordinates of both feet. Figure 2A illustrates the vertical trajectory of the COP for each foot’s movement. The difference in COP values between the left and right foot, known as the differential COP, was calculated, as shown in Figure 2B.

**FIGURE 3:**
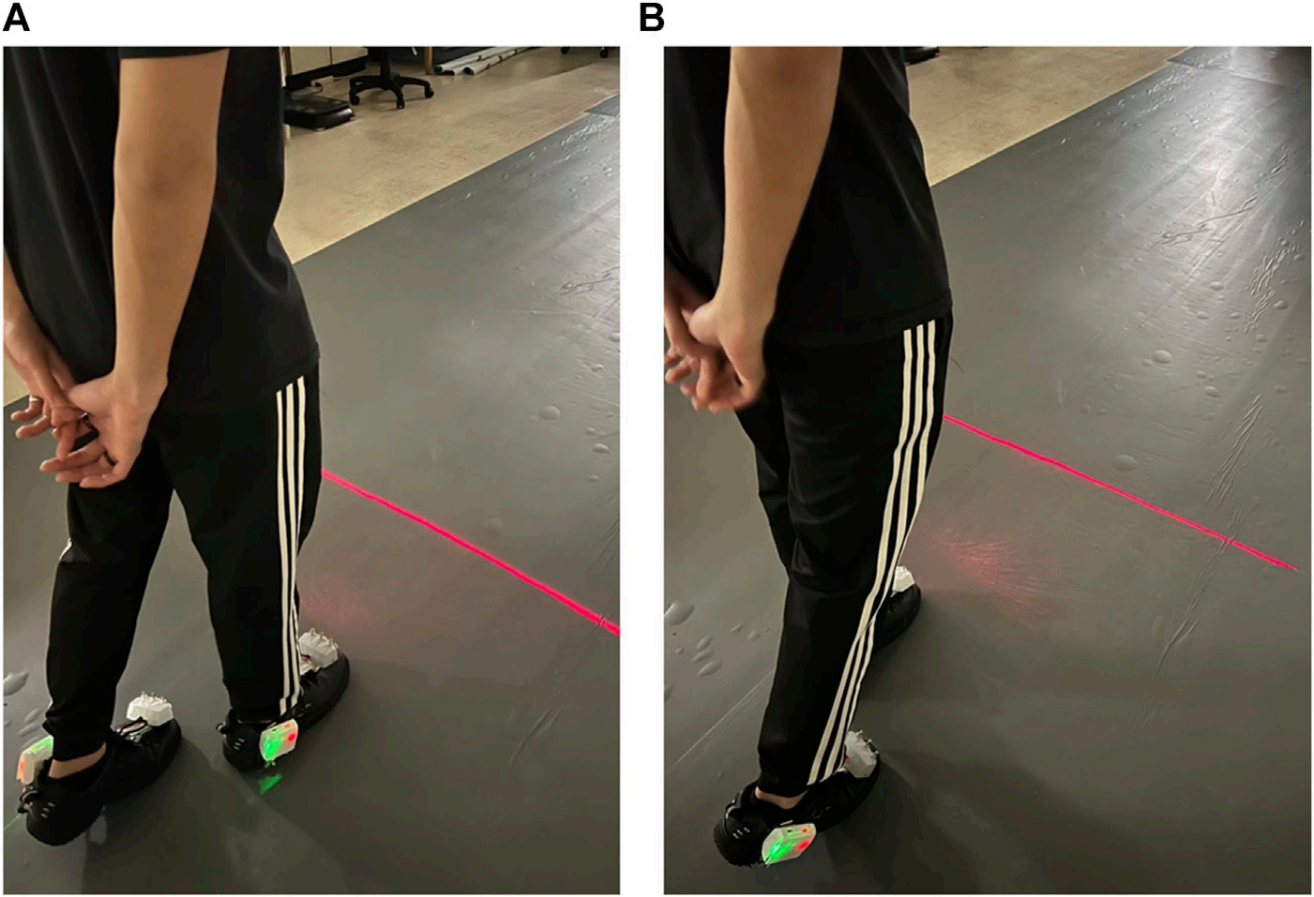
Laser-light visual cueing for Individuals with Parkinson’s Disease: **(A)** The laser-light device on the top cap of the right shoe projects a cueing line for the left foot to step over; **(B)** the laser-light device on the top cap of the left shoe projects a cueing line for the right foot to step over.

When a heel-strike occurs on the right foot, the right COPy descends towards the heel region, while the left COPy rises towards the toe area. This leads to a continuous increase in the differential COP. Once this differential COP exceeds a predetermined threshold, the laser cueing on the right shoe is initiated, offering a visual cue for the left foot, as shown in Figure 3A. Subsequently, the left foot progresses through its toe-off and swing phases, as indicated in Figure 2A.

Conversely, when a heel-strike occurs on the left foot, the left COPy descends towards the heel region, while the right COPy rises towards the toe area. This results in a continuous decrease in the differential COP. When the differential COP falls below another predetermined threshold, the laser cueing on the left shoe is activated, providing a visual cue for the right foot, as depicted in Figure 3B. This is succeeded by the right foot’s toe-off phase and swing phase.

### 2.2 Subjects and gait experiment

Twenty-six individuals diagnosed with PD, reporting episodes of FOG characterized by the sensation of their feet getting glued to the floor while walking, making a turn, or when trying to initiate walking, were recruited from the neurological department for participation in gait experiments. These episodes occurred during either on-medication or off-medication periods. The inclusion criteria include idiopathic PD with Hoehn and Yahr stage (H&Y) one to four, stable medication usage (no medication dosage changes 3 months before enrolling in the study), and be able to walk at least 6 m indoors without using assistive devices. Subjects were excluded if they had a diagnosis of central nervous system diseases other than PD, including cardiopulmonary vascular disease, orthopedic disease, audiovisual sensory disease, symptoms of dementia, dyskinesia, or were undergoing deep brain stimulation. All participants did not have visual dysfunction, meeting one of the inclusion criteria, and allowing them to see the laser-light cues in front of them.

To optimize motor control during the trials, they took their dopaminergic medications approximately 2–3 h prior to the experiment. Every participant completed two sets of walking back and forth on a walkway that measured 600 cm long and 90 cm wide. Both gait trials were conducted with and without visual cueing, and the order of these conditions was counterbalanced among the participants. However, four participants eventually dropped out of the experiment as they were unable to complete the gait trials. One participant dropped out due to medication degradation, while the others lacked the strength to continue walking. Ten out of the inclusive participants simultaneously performed the standard walking tests on an electronic walkway known as GAITRite, developed by CIR Systems Inc. in Sparta, NJ, United States of America. The protocol of this study was approved by the Research Ethics Committee of the Chang Gung Medical Foundation (IRB#201900664B0) in accordance with the Helsinki Declaration, and written informed consent was obtained from all participants.

The occurrence of FOG during gait trials was identified through post-session videos. The videos recorded during the off-cueing trials were reviewed and divided into two groups: participants exhibiting gait issues, such as shuffling steps and FOG while walking, were assigned to the worse-gait group, while those without these issues were categorized as the well-gait group. All participants from both groups were confirmed to be in an on-medication status. The aforementioned assessments were conducted through consensus among the expert panel of authors, which includes a neurologist, a physical therapist, and a medical engineering expert. Demographic data for both groups are summarized in Table 1. It is evident that the worse-gait group had higher scores in self-evaluation of activities of daily living (UPDRS II), clinician-scored motor evaluation (UPDRS III), and symptom progression (Hoehn and Yahr) compared to the well-gait group.

**TABLE 1:** The demographic data of individuals with Parkinson’s disease.

	Well-gait group (N = 12)	Worse-gait group (N = 10)	*p*-value
Age, years	69.1 ± 5.3	67.0 ± 6.8	0.4521
Gender, M/F	10/2	7/3	0.4831
Disease duration, years	11.04 ± 5.75	14.50 ± 11.51	0.3707
UPDRS II	9.0 ± 6.2	21.8 ± 5.9	0.0021
UPDRS III	26.3 ± 7.4	46.5 ± 5.2	0.0001
Hoehn and Yahr	2.50 ± 0.27	3.17 ± 0.41	0.0030
MMSE	29.0 ± 1.2	25.2 ± 5.8	0.1119

### 2.3 Gait parameters

Four temporal gait parameters were derived based on foot pressures. Firstly, a heel-strike event was identified when the heel pressure exceeded a specific threshold. This threshold was computed as the minimal heel pressure plus 10% of the max-min difference in heel pressure. Likewise, a toe-off event was detected when the toe pressure dropped below a designated threshold, which was determined by adding the minimal toe pressure to 10% of the max-min difference in toe pressure. Subsequently, the stride time was measured as the duration taken to complete a full stride cycle, from heel-strike to the next heel-strike of the same foot. Stance time was calculated as the duration from heel-strike to toe-off, while swing time was defined as the time from toe-off to heel-strike. Finally, the percentage of the stance and swing phases was determined by calculating their ratios relative to the stride time.

The stride length was determined using inertial data, where the swing angle was calculated by integrating the angular velocity in the swing direction over the swing time. The stride length was then computed by multiplying the leg length with the swing angle. To validate the estimated stride length, 598 gait cycles were identified simultaneously using both the GAITrite electronic walkway and foot inertial data. The electronic walkway provided an accurate measurement of the distances between consecutive left or right steps, serving as a reliable reference for stride length. By employing linear least-squares regression analysis, the relationship between the stride lengths derived from the inertial-based method and the walkway-based method was examined to evaluate the feasibility of the inertial-based estimation. To identify influential outliers among the predictor variables in the least-square regression, Cook’s distance was employed. Once the outliers were identified, they were excluded from the analysis. Subsequently, a linear regression model was constructed to calibrate the estimated stride length using the measurements obtained from the inertial data, taking into account the removed outliers.

### 2.4 Center of pressures trajectory derived parameters

A high-resolution map of foot pressure distribution was generated using a conditional generative adversarial network (GAN) based on measurements from eleven foot pressures. The GAN model was developed using measurements obtained from a customized eleven-FSR insole and Pedar-X insole, which featured a 99-point pressure sensor pad covering the entire plantar surface of the foot, provided by Novel GmbH in Munich, Germany (Liang, 2022). The COP for each foot was calculated from the reconstructed foot pressure distribution by summing the products of the reconstructed foot pressures and their corresponding x and y positions, and dividing by the sum of the reconstructed foot pressures:

$$\hat{\text{COP}}x\left(i\right)=\frac{\sum \sum \left({\hat{fp}}_{m,n}\left(i\right)*x_{m,n}\right)}{\sum \sum {\hat{fp}}_{m,n}\left(i\right)}$$

$$\hat{\text{COP}}y\left(i\right)=\frac{\sum \sum \left({\hat{fp}}_{m,n}\left(i\right)*y_{m,n}\right)}{\sum \sum {\hat{fp}}_{m,n}\left(i\right)}$$

where *x*_*m,n*_ and *y*_*m. n*_ represent the coordinates of the reconstructed foot pressures with respect to the local reference frame.

**FIGURE 4:**
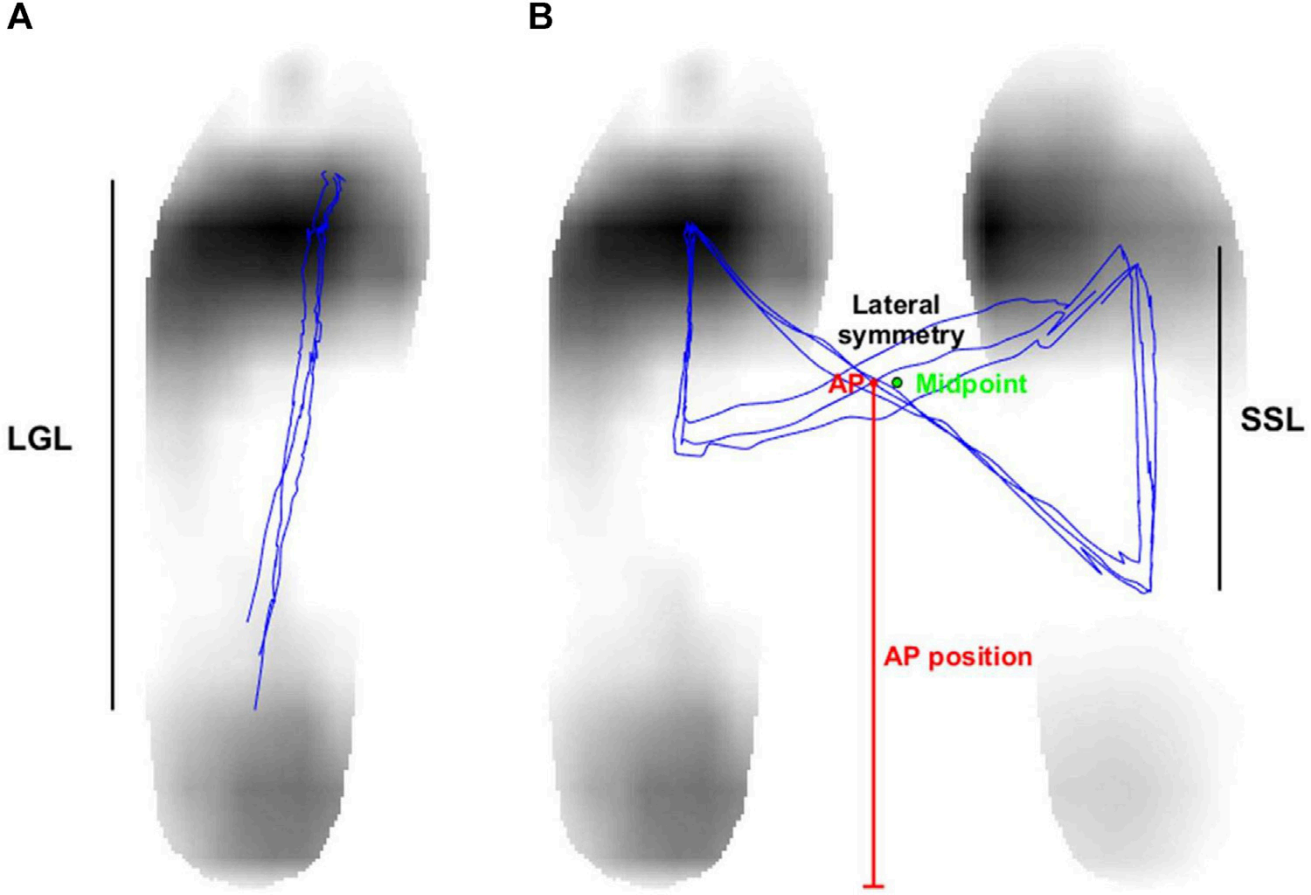
**(A)** The length of gait line (LGL) is characterized as the length of the center of pressure’s (COP) changed position during the stance time, which is the duration between the left foot’s heel-strike and toe-off. **(B)** The single support length (SSL) is described as the distance covered by the COP’s movement during the single support time. In addition, the AP position is identified as the vertical distance between the COP intersection point and the foot’s bottom. The horizontal distance between the COP intersection point and the midpoint of both feet determines the lateral symmetry.

The gait line in each gait cycle was established by analyzing the COP trajectory during the stance time, which is the interval between heel-strike and toe-off in either the left or right foot (Yoo et al., 2017). This allowed the calculation of length of gait line in both feet, defined as the length of the changed position of the COP during the stance time as depicted in Figure 4A.

The COP for both feet was calculated by combining the COPs of the left foot and the right foot using a weighted sum, with the horizontal distance between the two feet standardized to 15 cm. A butterfly diagram was produced by analyzing the COP of both feet, as illustrated in Figure 4B. The single support length (SSL) was subsequently derived from this diagram. The SSL refers to the length of the COP’s displacement during the single support time, which is when one foot is in the stance phase and the other is in the swing phase (Yoo et al., 2017). Double support time is defined as the period when both feet maintain contact with the ground throughout the entire gait cycle. Finally, the percentage of the single support and double support phases was determined by calculating their ratios relative to the stride time. In addition, we assessed the anterior-posterior (AP) position and lateral symmetry by upsampling the COP of both feet by a factor of 10 and identifying the central point where the COP trajectory intersects. The vertical distance between the COP intersection point and the foot’s bottom determined the AP position, while the horizontal distance between the COP intersection point and the midpoint of both feet measured the lateral symmetry (Yoo et al., 2017).

### 2.5 Freezing parameters

Moore et al. introduced the concept of a freeze index (FI) based on vertical shank acceleration, calculated as the ratio of power between a locomotion band (0.5–3 Hz) and a freeze band (3–8 Hz) (Moore et al., 2008). Subsequently, Moore et al. demonstrated higher FIs during episodes of FOG using accelerometry on the lumbar, thigh, shank, and foot. In our study, we analyzed the kinematics of foot accelerations around the heel in the mediolateral, anteroposterior, and superoinferior directions to quantify FOG characteristics. (Moore et al., 2013). In our study, we analyzed the kinematics of foot accelerations around the heel in the mediolateral direction to quantify FOG characteristics.

**FIGURE 5:**
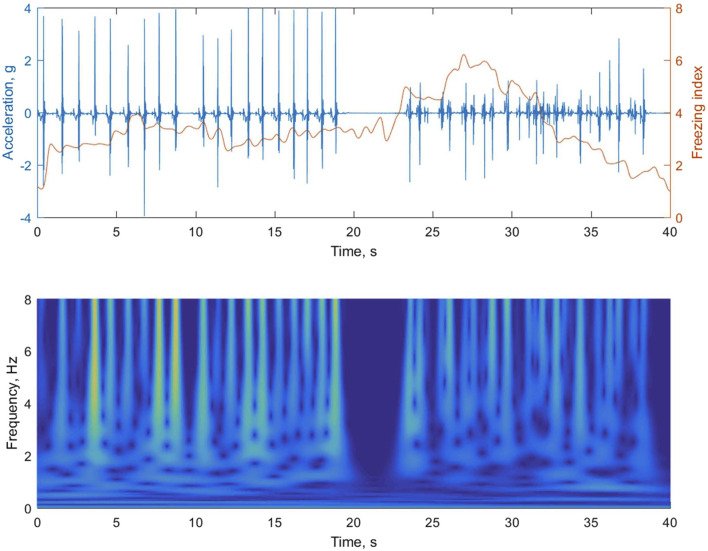
The upper panel of the figure shows the foot accelerations during regular walking followed by an episode of gait freezing. In the lower panel, the magnitude of the time-frequency representation of the acceleration signal is obtained using continuous wavelet transform (CWT). The instantaneous freezing index is calculated by summing the absolute square of the CWT values for both a locomotion band (0.5–3 Hz) and a freeze band (3–8 Hz), and then determining the ratio between them in the upper panel.

In our analysis of foot accelerations, we utilized continuous wavelet transform (CWT) *using the Morlet wavelet* to examine the time-frequency characteristics. CWT offers several advantages, as it employs wavelet functions of varying scales. Smaller-scale wavelet functions are able to capture rapid changes in the signal, enhancing the time resolution for high-frequency components. Conversely, larger-scale wavelet functions are wide enough to encompass slow-varying characteristics, resulting in improved frequency resolution for low-frequency components. Figure 5 depicts how the walking rhythm is accurately represented by the larger-scale wavelets in a lower frequency band (less than 3 Hz), but this representation diminishes during subsequent episodes of FOG. Conversely, the smaller-scale wavelets in a higher frequency band (greater than 3 Hz) effectively capture the rapidly changing accelerations caused by ground impacts during walking or gait freezing.

The instantaneous FI was obtained by summing the absolute square of the CWT values for both a locomotion band and a freeze band, and then calculating the ratio between them. The calculation was performed using a time window. In each gait trial, the instantaneous FI during walking was compared to a threshold. If the FI exceeded the threshold, it was classified as a freeze-prone point. The portion of periods prone to freezing was calculated as the ratio of the number of classified freeze-prone points to the total number of instantaneous FI values. The threshold was established based on three values: 2, 2.5, and 3, demonstrating effective performance in quantifying the freeze-prone period, as elaborated in the Results section. Additionally, the instantaneous FI values were averaged to obtain a representative FI value.

## 3 Results

**FIGURE 6:**
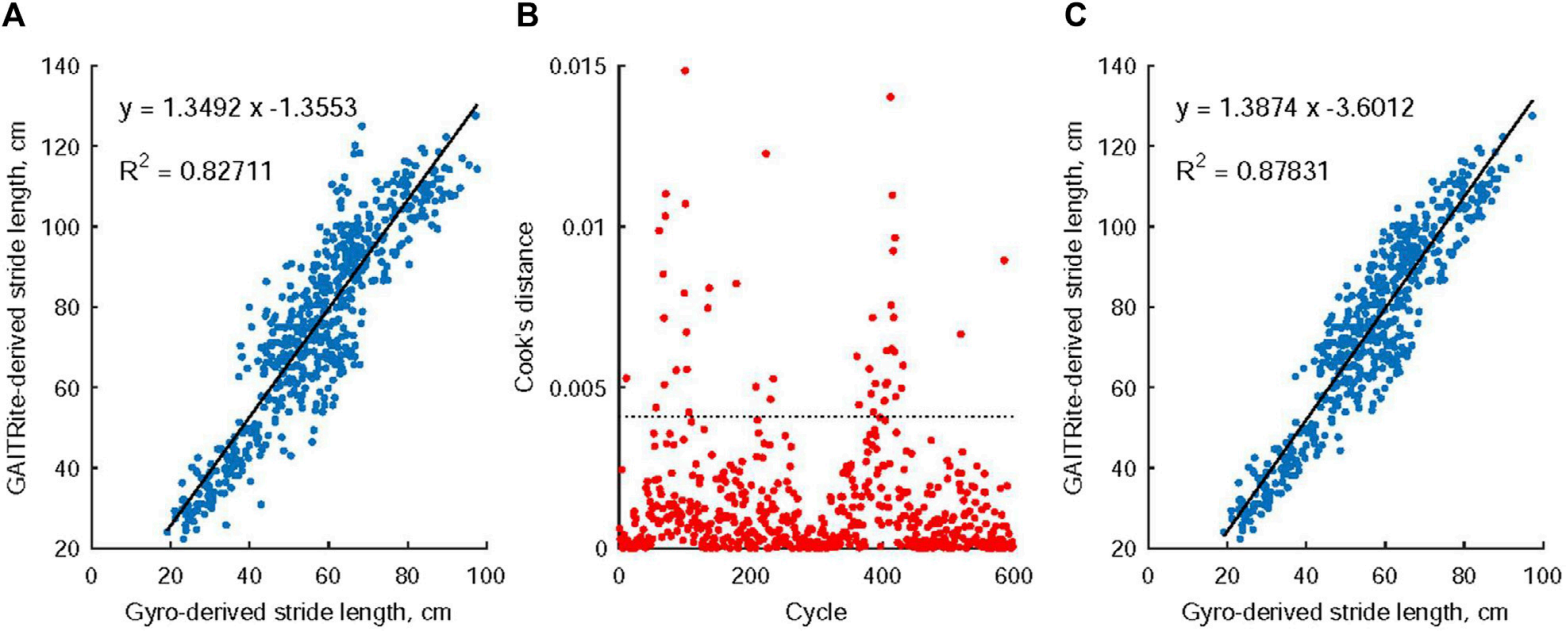
The relationship between stride length measurements obtained from the GAITRite electronic walkway and those estimated through angular velocity analysis during the swing phase of walking. Notably, in **(A)**, a linear connection was observed between these two stride measurements. Moving to **(B)**, 46 observations were identified as noteworthy outliers due to their Cook’s distance values exceeding 3 times the mean Cook’s distance value. Subsequently, **(C)** details the creation of the ultimate regression model, which was constructed using data that had these identified outliers removed.

Figure 6A presents a comparison between the stride length obtained from the GAITRite electronic walkway and the stride length estimated using angular velocity analysis during the swing phase of walking. A regression approach was applied to establish a relationship between these two stride measurements by fitting a linear trendline to the collected data points. To identify potential outliers among the 598 observations, a threshold (depicted as the dashed line in Figure 6B) was set at 3 times the mean value of Cook’s distance. Notably, 46 observations were identified as significant outliers based on their higher Cook’s distance values (Figure 6B). Consequently, the final regression model was developed using data that excluded these outliers (Figure 6C), and this model was then employed as a predictive tool to estimate stride length for all identified gait cycles.

Table 2 displays the outcomes of the gait parameter analysis conducted on individuals with PD while walking both forward and backward, both with and without laser-light cues. In the absence of cues, the group exhibiting poorer gait demonstrated significantly shorter stride lengths, significantly larger percentages of stance phase, and significantly smaller percentages of swing phase on both their affected and unaffected sides compared to the well-gait group. When presented with visual cues, the well-gait group exhibited significant changes in various parameters. These changes encompassed substantial increases in both stride time and the percentage of the stance phase, coupled with a notable reduction in stride length on both the affected and unaffected sides. In contrast, the incorporation of visual cues led to a slight but significant decrease in the swing percentage of the affected foot in the worse-gait group. After analyzing the coefficient of variation of each gait parameter, no significant differences in gait variability were observed between the well-gait and worse-gait groups, nor between on-cueing and off-cueing conditions.

**TABLE 2:** The statistical results of gait parameters during forward and backward walking, comparing conditions with and without laser-light cueing in individuals with Parkinson’s disease.

	Well-gait group			Worse-gait group		
	Off-cueing	On-cueing	*p*-value	Off-cueing	On-cueing	*p*-value
Stride length, cm						
affected	88.32 ± 14.97	76.19 ± 13.01	**0.011**	48.80 ± 12.28*	48.86 ± 14.27*	0.977
unaffected	87.60 ± 15.62	78.04 ± 12.93	**0.049**	48.97 ± 12.37*	47.74 ± 13.13*	0.518
Stride time, s						
affected	1.478 ± 0.254	1.938 ± 0.329	**0.002**	1.647 ± 0.929	1.533 ± 0.458*	0.653
unaffected	1.513 ± 0.288	1.952 ± 0.307	**0.009**	2.255 ± 2.796	1.520 ± 0.433*	0.409
Stance phase, %						
affected	61.57 ± 2.95	63.89 ± 3.37	**0.046**	67.90 ± 7.56*	68.80 ± 6.35*	0.240
unaffected	61.81 ± 3.04	64.51 ± 5.30	**0.012**	69.39 ± 6.99*	70.16 ± 7.70	0.335
Swing phase, %						
affected	37.95 ± 2.73	36.55 ± 2.95	0.187	32.84 ± 6.45*	31.49 ± 6.32*	**0.049**
unaffected	37.99 ± 2.49	35.54 ± 5.14	0.054	31.04 ± 7.54*	30.01 ± 7.96	0.144

The data were expressed as mean ± standard deviation. To determine significant differences, paired t-tests were performed to compare walks with and without cueing with statistical significance set at *p* < 0.05, denoted by bold. Likewise, independent t-tests were conducted to compare the well- and worse-gait groups, and the statistical significance was indicated by *.

The results of the COP parameter analysis for individuals with PD walking forth and back, with and without laser-light cues, are presented in Table 3. In both the well-gait and worse-gait groups, the length of single support on the affected side was shorter than that on the unaffected side. In the off-cueing condition, the worse-gait group exhibited several noteworthy differences compared to the well-gait group. These distinctions included significantly shorter single support lengths and smaller percentages of single support phases on both the affected and unaffected sides. Additionally, there was a larger percentage of double support phase, along with larger lateral symmetry in the COP’s butterfly intersection.

**TABLE 3:** The statistical results of center of pressures trajectory derived parameters during forward and backward walking, comparing conditions with and without laser-light cueing in individuals with Parkinson’s disease.

	Well-gait group			Worse-gait group		
	Off-cueing	On-cueing	*p*-value	Off-cueing	On-cueing	*p*-value
Length of gait line, cm						
affected	21.59 ± 3.51	28.51 ± 6.64	**0.001**	22.64 ± 4.90	24.57 ± 7.60	0.165
unaffected	24.80 ± 3.19 †	30.94 ± 5.39	**<0.001**	24.67 ± 6.77	26.90 ± 7.36	**0.026**
Single support length, cm						
affected	16.15 ± 2.90	14.68 ± 3.75	0.164	10.78 ± 4.23*	9.93 ± 4.57*	0.226
unaffected	18.80 ± 2.85 †	18.86 ± 6.38 †	0.954	14.42 ± 3.87* †	13.79 ± 3.80* †	0.570
Single support phase, %						
affected	37.67 ± 3.13	34.11 ± 7.25	**0.036**	30.16 ± 8.77*	28.91 ± 9.29	**0.045**
unaffected	37.51 ± 2.82	35.10 ± 4.60	0.091	33.27 ± 5.56*	32.10 ± 5.51	0.393
Double support phase, %	24.90 ± 4.21	28.59 ± 8.15	**0.048**	35.73 ± 11.21*	37.57 ± 9.20*	0.339
Lateral asymmetry, cm	0.718 ± 0.601	1.258 ± 0.738	**0.012**	1.519 ± 0.758*	1.574 ± 0.731	0.688
AP position, cm	14.95 ± 1.02	15.13 ± 1.37	0.611	14.83 ± 1.75	14.91 ± 1.55	0.790

The data were expressed as mean ± standard deviation. To determine significant differences, paired t-tests were performed to compare walks with and without cueing, as well as affected and unaffected sides, with statistical significance set at *p* < 0.05. Significant differences were denoted by bold and †, respectively. Likewise, independent t-tests were conducted to compare the well- and worse-gait groups, and the statistical significance was indicated by *.

Upon the introduction of visual cues, the well-gait group displayed an extension in the length of the gait line on both the affected and unaffected sides, an elevation in the percentage of double support phase, and a reduction in the percentage of single support phase on the affected side, accompanied by an increase in lateral symmetry. Conversely, the worse-gait group exhibited an increase in the length of the gait line on the unaffected side and a reduction in the percentage of single support phase on the affected side in the presence of visual cues.

We performed statistical tests to compare the average FIs obtained from the on-cueing and off-cueing conditions, using instantaneous FI calculations with window sizes of 3, 4, 5, and 6 s. Our findings revealed that visual laser-light cueing significantly decreased the average FI only in the affected foot of the worse-gait group. Additionally, we conducted statistical tests to compare freeze-prone percentages between the on-cueing and off-cueing conditions, using different thresholds to determine freeze-prone points. Our results showed that visual laser-light cueing significantly decreased the freeze-prone percentage only in the affected foot of the worse-gait group. This effect was observed when thresholds of 2, 2.5, and 3 were used, and window sizes were set at 5 and 6 s. Hence, for our analysis, we employed a window size of 6 s and a threshold of 3.

The statistical results of the comparisons between PD individuals walking with and without laser-light cueing are presented in Table 4. In comparison to the well-gait group, the worse-gait group displayed significantly higher average FI values on both their affected and unaffected sides, along with a greater proportion of periods prone to freezing on the affected side. However, when the worse-gait group incorporated visual cues into their movements, notable changes occurred. They experienced a significant reduction in the average FI value on the affected side while executing a U-turn, while there was a substantial decrease in the percentage of periods prone to freezing on the affected side during straight walking.

**TABLE 4:** The statistical results of freeze related parameters during walking, comparing conditions with and without laser-light cueing in individuals with Parkinson’s disease.

	Well-gait group			Worse-gait group		
	Off-cueing	On-cueing	*p*-value	Off-cueing	On-cueing	*p*-value
Affected side						
Average FI						
Straight	0.854 ± 0.303	0.821 ± 0.194	0.581	1.801 ± 0.788*	1.532 ± 0.555*	0.060
U-turn	0.816 ± 0.283	0.956 ± 0.484	0.129	1.721 ± 0.829*	1.362 ± 0.644	**0.036**
Freeze-prone portion, %						
Straight	0.11 ± 0.38	0 ± 0	0.339	11.30 ± 15.72*	2.54 ± 4.76	**0.049**
U-turn	0.04 ± 0.12	0.77 ± 1.80	0.190	12.07 ± 22.48	3.30 ± 8.39	0.104
Unaffected side						
Average FI						
Straight	0.882 ± 0.559	0.940 ± 0.524	0.455	1.762 ± 0.971*	1.448 ± 0.591*	0.219
U-turn	0.743 ± 0.241	0.801 ± 0.282	0.347	1.758 ± 1.083*	1.468 ± 0.549*	0.187
Freeze-prone portion, %						
Straight	0.81 ± 1.61	0.52 ± 1.81	0.671	9.94 ± 18.95	3.56 ± 7.47	0.228
U-turn	0 ± 0	0 ± 0		10.85 ± 23.61	4.80 ± 10.28	0.207

The average freeze index (FI) and freezing-prone portion are reported as mean ± standard deviation. To determine significant differences, paired t-tests were performed to compare walks with and without cueing with statistical significance set at *p* < 0.05, denoted by bold. Likewise, independent t-tests were conducted to compare the well- and worse-gait groups, and the statistical significance was indicated by *.

**FIGURE 7:**
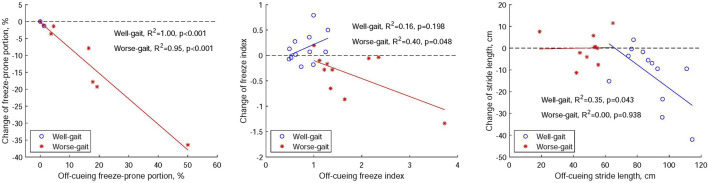
Relationships between off-cueing parameters (freeze-prone portion, the averaged freeze index, and stride length) and the delta change (on cueing - off cueing) in these parameters after the application of visual cues within both Parkinson’s disease groups, distinguishing between those with well-gait and worse-gait characteristics.

Figure 7 illustrates the relationships between off-cueing parameters and the delta change (on cueing - off cueing) in these parameters following the utilization of visual cues in both the well-gait and worse-gait groups. Within the worse-gait group, the percentage of the freeze-prone portion during straight walking exhibited the strongest association with gait improvement when visual cues were employed, and this association decreased but remained significant when assessing gait improvement using the averaged FI during turning. However, only half of the worse-gait participants demonstrated an increase in stride length during straight walking. In contrast, the well-gait group exhibited a reduction in stride length with visual cues, indicating a negative association with gait improvement.

Upon reviewing the videos to identify FOG episodes, we noted the absence of FOG experiences in the participants of the well-gait group. However, FOG was observed in three participants within the worse-gait group, particularly during the off-cueing condition. They experienced 9 FOG episodes with a percentage of time frozen at 45.56%, 1 episode with 15.9%, and 1 episode with 8.60%, respectively. The application of visual cues resulted in an improvement in the freezing episodes for these participants. The first participant exhibited a reduction to 3 FOG episodes and a percentage of time frozen at 11.11%, while the remaining two participants did not experience any FOG with the use of visual cues. These improvements in FOG were accompanied by an increase in stride length for the affected foot for all three participants. Specifically, the first participant showed an increase from 18.9 to 26.5 cm, the second from 53.7 to 54.4 cm, and the third from 52.6 to 58.3 cm, respectively.

## 4 Discussion

In this study, individuals with PD were recruited to participate in gait trials conducted under both off-cueing and on-cueing conditions. The recruited participants were divided into two groups: the well-gait group and the worse-gait group. The well-gait group exhibited better abilities in performing daily activities and motor execution, and showed a lower degree of movement disorders in comparison to the worse-gait group. This distinction was supported by their UPDRS II, III, and Hoehn and Yahr grades, as presented in Table 1.

In the off-cueing condition, the well-gait group demonstrated a longer stride length compared to the worse-gait group. Nonetheless, our findings unveiled distinct responses to visual cueing between the well-gait group and the worse-gait group. In the well-gait group, the introduction of visual cueing resulted in a reduction in stride length, an elongation in stride time, and an increase in percentages of stance and double support phases during walking. These changes can be attributed to the fact that the well-gait participants already exhibited proficient walking patterns. The specific setup of the visual cues, where the laser beam was positioned 30–35 cm away from the front foot, guided participants to take shorter steps when crossing the beam as compared to the off-cueing condition. This suggests that visual cues effectively regulated the participants’ gait, resulting in a more cautious and stable walking pattern.

In the current study, the group with better gait performance showed a lower percentage of stance phase and a higher percentage of swing phase in comparison to the group with worse gait. This trend aligns with results reported in earlier studies (Farashi, 2021; Zanardi et al., 2021). Notably, when visual cues were introduced, the better gait group showed an increase in the percentage of stance phase. The explanation for the negative effects observed in the better gait group has been discussed, linking them to the shift from the naturally automated walking process to a compensatory cortical-controlled gait influenced by visual cues. Importantly, no adverse effects are noted in the worse gait group.

Moreover, the worse-gait group exhibited a smaller percentage of the single support phase and a larger double support phase compared to the well-gait group. The lengthening of the double support phase may be associated with increased postural instability and fear of falling due to their lower gait quality (Zanardi et al., 2021; Costa et al., 2022). Nevertheless, the introduction of visual cues resulted in only a slight reduction in the percentage of single support phase within the worse-gait group. It is noteworthy that a single session of visual cue training is insufficient to induce substantial changes in gait patterns, such as altering the percentage of single and double support phases. Instead, it is likely to shift the gait from automatic control towards cognitive-controlled gait.

In the literature, individuals with PD have exhibited greater gait variability compared to age-matched healthy subjects, indicating diminished automatic control of walking (Hausdorff et al., 1998). Gait variability became more pronounced in PD individuals when engaged in cognitively demanding tasks during walking, highlighting the substantial influence of attention on gait (Hausdorff et al., 2003). A comprehensive review has identified varied responses among individuals with PD to rhythmic auditory stimulation (Harrison and Earhart, 2023). Notably, gait variability significantly decreased in PD individuals who employed an attention strategy—specifically, focusing on taking larger steps—while walking with rhythmic auditory cueing (Baker et al., 2008). In terms of the impact of visual cueing on gait variability in individuals with PD, a study revealed an enhancement in stride time variability through the utilization of visual cues, specifically by placing transverse lines on the floor (Graham et al., 2023). Contrastingly, the use of visual cues through a carpet with horizontal lines resulted in an increase in the variability of both step width and step time (Vitório et al., 2014). However, in the current study, no noteworthy changes in gait variability were observed during straight walking with visual cues. For future investigations, it is advisable to incorporate a larger number of strides to thoroughly explore the potential effects of visual cueing on gait variability in individuals with PD.

In the research utilizing wearable visual cueing, mounting the cueing device on the chest or waist led to an increase in stride length compared to the off-cueing condition (Lewis et al., 2000; Suputtitada et al., 2022; Zhang et al., 2023). However, no significant changes were observed when the cueing devices were attached to the shoes (Barthel et al., 2018), which aligns with the results seen in the worse-gait group in our study. This can be attributed to the fact that a laser-light device attached to the human body at a higher height, such as the chest or waist, can be adjusted to project visual cues over a longer distance from the individual’s toes, approximately 50 cm (Lewis et al., 2000) or 110% of the average step length (Zhang et al., 2023). This approach closely approximates the distances between transverse lines or inverted sticks in traditional visual cues, which typically range from 45 to 50 cm (Azulay et al., 1999; Lewis et al., 2000; Lee et al., 2012) or correspond to about 40% of an individual’s height (Suteerawattananon et al., 2004). Consequently, this adjustment led to an increase in stride length when visual cues were applied. It is worth noting that a study has employed visual cues with different distances between the cues and the participants (Wu et al., 2020). To gain further insights and clarify this issue, it may be beneficial to explore the possibility of reconfiguring the lighting mechanism to offer visual cues at various distances for clinical assessments. This could help researchers better understand the impact of cueing distance on gait parameters and potentially optimize cueing strategies for individuals with different gait profiles.

Regarding the impact of visual cueing on FOG episodes in individuals with PD, various studies have reported differing outcomes. For instance, Bryant et al. demonstrated a reduction in the number of freezing episodes by utilizing laser-light visual cueing from a cane (Bryant et al., 2010). Velik et al. reported a decrease in both the duration and number of freezing episodes when visual cueing projected from the chest was applied (Velik et al., 2012). Barthel et al. found that laser-light visual cueing from shoes led to a reduction in FOG occurrences (Barthel et al., 2018). However, it is worth noting that other studies, such as the one conducted by Bunting-Perry et al., which utilized visual cues emitted from ankle-level placements on a four-wheeled rolling walker, did not report significant effects on the occurrences of FOG episodes (Bunting-Perry et al., 2013). In this study, we employed acceleration-derived freezing indices, a method previously utilized for detecting FOG episodes (Moore et al., 2008; Moore et al., 2013) and distinguishing FOG-vulnerable gait from normal gait (Chen et al., 2019). Additionally, the method was applied to assess the improvement of FOG through auditory cueing and somatosensory cueing (Mancini et al., 2018). We validated the FI using post-session videos. During episodes of FOG, the corresponding FI was observed to be higher compared to when participants were in a better walking state. These scenarios are notably pronounced within the worse-gait group. As a result, visual cueing effectively reduced both the FI and the freeze-prone portion, as determined from foot accelerations on the affected side in the worse-gait group.

In general, while the impact on gait parameters is not extensively documented, the efficacy of visual cues is particularly pronounced in mitigating freezing within the group with worse gait. This aligns with outcomes observed in a prior study utilizing shoe-based visual cueing (Barthel et al., 2018). Our findings highlight improvements in variables associated with freezing, emphasizing the importance of patient selection in the clinical application of visual cueing.

Individuals with PD often exhibit an asymmetric butterfly diagram in the COP trajectory of their feet when compared to normal subjects. Two commonly employed metrics for quantifying this asymmetry are single support length (SSL) and lateral symmetry (LS). SSL is measured for both feet during their single support phase, while LS is the horizontal distance between the COP intersection point and the midpoint of both feet. In this study, it was observed that the affected foot had a shorter SSL than the unaffected foot in both the well-gait and worse-gait groups. This finding aligns with previous research (Shin and Ahn, 2020). Furthermore, the worse-gait group exhibited shorter SSL values on both the affected and unaffected sides when compared to the well-gait group. This observation is consistent with the notion that individuals with PD tend to have shorter SSL values than normal subjects, as demonstrated in prior studies (Shin and Ahn, 2020).

On the other hand, the worse-gait group displayed higher values of LS compared to the well-gait group in the absence of visual cueing. This observation aligns with the established notion that individuals with PD often exhibit greater LS values than their healthy counterparts (Shin and Ahn, 2020). Notably, the introduction of visual cueing did not induce a significant change in LS for the worse-gait group. It is noteworthy that visual cueing did not lead to significant changes in either LS or SSL in individuals with PD, as has been previously demonstrated in other investigation (Zhang et al., 2023). Conversely, the utilization of visual cueing did lead to an increase in LS for the well-gait group. Given that the well-gait participants already demonstrated proficient walking patterns, the introduction of visual cueing appeared to force them into an unnatural walking gait in comparison to the off-cueing condition. This may explain the observed increase in LS in the on-cueing condition for the well-gait group.

Unlike the SSL measured exclusively during the single support phase, the length of gait line (LGL) is determined by the trajectory of the dynamic COP during the stance phase of one foot, encompassing both single stance and double stance phases. The introduction of visual cueing led to in an increase in the LGL on both the affected and unaffected sides in the well-gait group, as well as an increase in the LGL of the unaffected foot in the worse-gait group. This augmentation in LGL can be attributed to the extended stance phase facilitated by visual cueing.

In this study, individuals with PD participated in gait trials approximately 2–3 h after taking their medications, regardless of whether they belonged to the well-gait or worse-gait group. All participants from both groups were confirmed to be in the on-medication status through consensus among the expert panel of authors. This condition allowed individuals to walk while maintaining motor control, closely resembling daily-life walking situations, which is a common approach in most research due to the challenges presented by severe gait impairment during the off-medication phase, which could hinder the execution of gait trials.

Notably, the influence of visual cueing might differ between the on-medication and off-medication states. Research indicates that using a cane with visual cues led to an increase in stride length in both on-medication and off-medication states, but it reduced the number of freezing episodes only in the off-medication state (Bryant et al., 2010). Another study revealed a decrease in the number of FOG episodes when shoes-based visual cueing was applied in both on-medication and off-medication conditions, but it reduced the percentage of time spent frozen solely in the off-medication state (Barthel et al., 2018). Utilizing the integration of foot pressure and inertial sensing technology in our visual cueing shoes, our proposed device can be adapted for use in non-laboratory settings. This adaptation is particularly pertinent when evaluating the gait of individuals with PD and assessing the influence of laser-light visual cueing on gait in the off-medication condition, which closely resembles real-life scenarios.

Our study specifically investigates the immediate impact of visual cueing on gait adaptation and freezing during walking within the comprehensive group of participants with PD. The assessment of activities of daily living (ADL) relied solely on participants’ self-evaluation, as defined by UPDRS II, distinct from the ADL scale measuring effort and dependence on others for daily chores. As our system evolves for real-world implementation, expanding its application to clinical training demonstrates its potential effectiveness in improving daily-life activities through the utilization of visual cueing.

In the present study, we evaluated the effects of laser-light visual cueing on walking by assessing gait and freezing parameters. One limitation of our approach is the absence of self-report assessments regarding the device’s usability from each participant. Moreover, the characterization of FOG was solely based on self-reports, determining whether individuals perceived their feet as glued to the floor while walking, turning, or initiating walking, during either on-medication or off-medication periods, without employing a new Freezing of Gait Questionnaire for scoring.

## 5 Conclusion

We designed laser-light visual shoes to generate interleaved visual cues on the floor for the left and right feet, while simultaneously recording foot inertial data and foot pressures. Notably, our proposed visual cueing system operates by presenting cues to one side at a time, thus avoiding any potential confusion arising from simultaneous presentation of two cueing lines. In our study, visual cueing induced an extension of both stride time and the percentage of stance phase, alongside a reduction in stride length for PD individuals with well gait. This was attributed to the limited projection distance achievable through the shoes. Conversely, visual cueing in PD individuals with worse gait resulted in a decrease in both freeze index and the portion of intervals prone to freezing episodes. For future clinical assessments conducted in non-laboratory settings, enhancing the lighting mechanism to offer visual cues at a greater distance, along with the integration of foot pressure and inertial sensing into the visual cueing shoes, could provide valuable insights into the effects of visual cueing on gait control in individuals with PD.

## Data Availability

The raw data supporting the conclusion of this article will be made available by the authors, without undue reservation.
